# Supplementary material for: Nociceptive sensory neurons promote CD8 T cell responses to HSV-1 infection
Source: Nat Commun. 2021 May 18;12:2936. doi: 10.1038/s41467-021-22841-6 (PMC8131384; doi:10.1038/s41467-021-22841-6)
Supplement: Supplementary file 1 — Supplementary Information [file 41467_2021_22841_MOESM1_ESM.pdf]

Supplementary information for

Nociceptive sensory neurons promote CD8 T cell responses to HSV-1 infection

Jessica Filtjens[1*], Anais Roger[1*], Linda Quatrini[1,2], Elisabeth Wieduwild[1], Jordi Gouilly[1], Guillaume Hoeffel[1], Rafaëlle Rossignol[1], Clara Daher[1,3], Guilhaume Debroas[1], Sandrine Henri[1], Claerwen M. Jones[4], Bernard Malissen[1], Laura K. Mackay[4], Aziz Moqrich[5], Francis R. Carbone[4] and Sophie Ugolini[1]

[1] Aix Marseille Univ, CNRS, INSERM, CIML, Centre d'Immunologie de Marseille-Luminy, Marseille, France

[2] Department of Immunology, IRCCS Bambino Gesù Children's Hospital, Rome, Italy

[3] Université de Paris, CNRS, Institut Cochin, INSERM, CNRS, F-75014 PARIS, France

[4] Department of Microbiology and Immunology, The University of Melbourne at The Peter Doherty Institute for Infection and Immunity, Melbourne, VIC, Australia

[5] Aix-Marseille-Université, CNRS, Institut de Biologie du Développement de, Marseille, France

[*] These authors contributed equally to this work

Correspondence: Sophie Ugolini

Centre d'Immunologie de Marseille Luminy

Parc Scientifique et Technologique de Luminy

Case 906- 13288 Marseille cedex 9- FRANCE

Phone: +33 (0)4 91 26 94 44

E-mail: ugolini@ciml.univ-mrs.fr

This PDF file includes :

Supplementary Figure 1

Supplementary Figure 2

Supplementary Figure 3

Supplementary Figure 4

Supplementary Figure 5

Supplementary Table 1

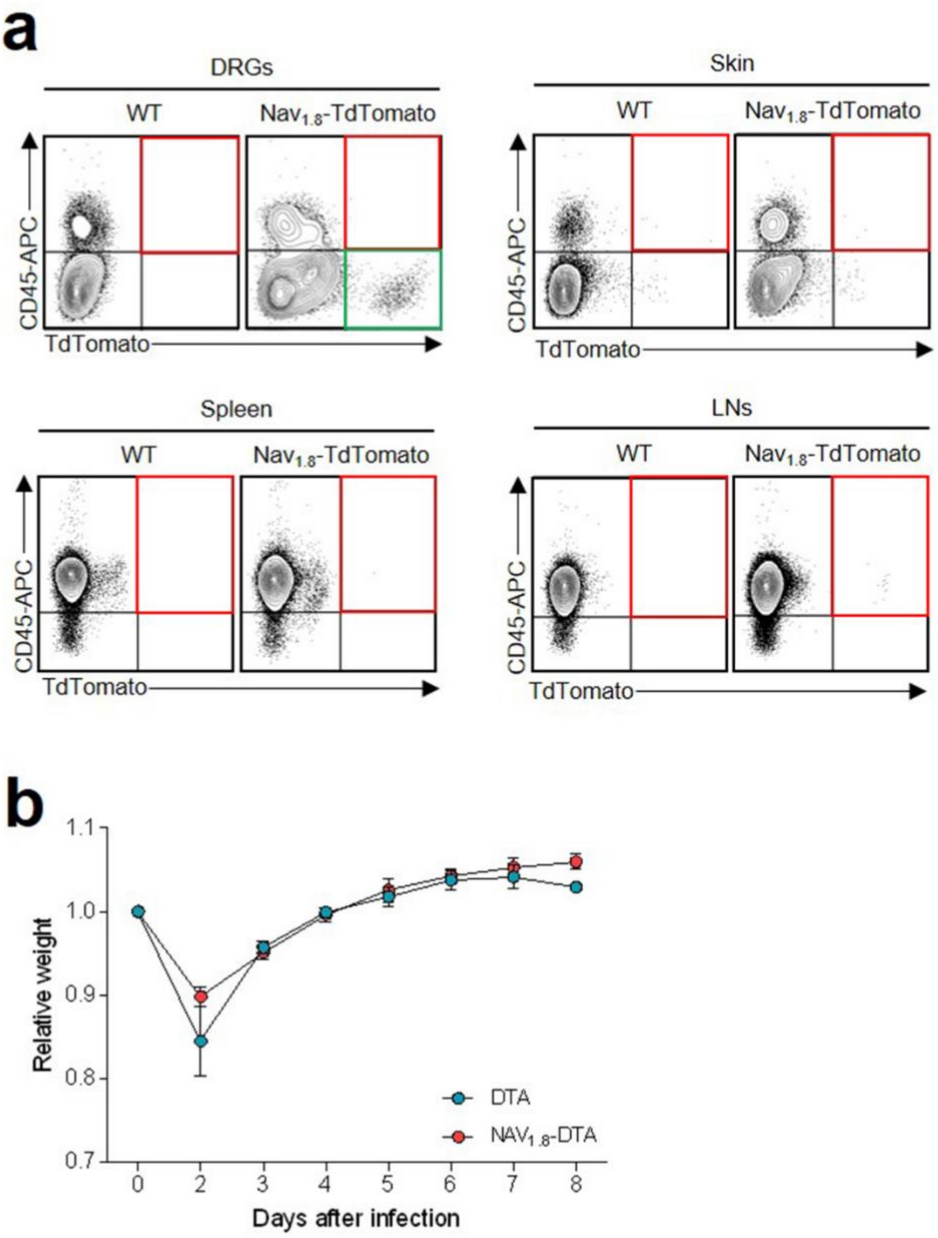

Supplementary figure 1. $Nav_{1.8}$ is expressed in neurons but not in other skin cell types. (a) Flow cytometry analysis of $Nav_{1.8}$-TdTomato mouse tissues after cell extraction and staining with an anti-CD45 antibody. Dot plots of DRGs, skin, spleen and lymph node (LN) cells are shown (n=1 mouse). **(b)** Relative weights of control DTA (blue) and $Nav_{1.8}$-DTA (red) mice infected with HSV-OVA-TK^- are presented from days 0 to 8 pi. Relative weights were calculated as a percentage of the original weight of the mice on day 0, (n=17-22 mice per group). The results shown are representative of three independent experiments. The data shown are the mean ± SEM.

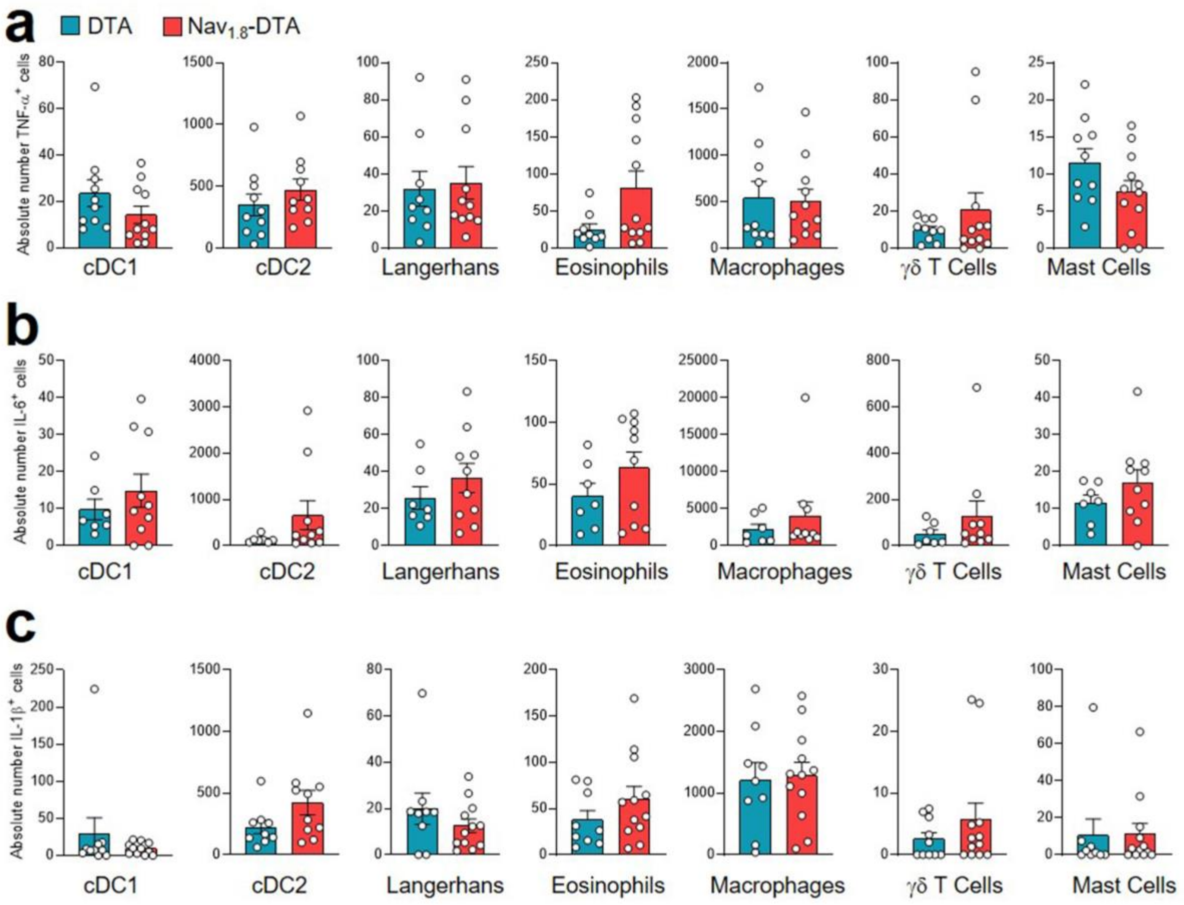

Supplementary figure 2. Cellular source of pro-inflammatory cytokines in the skin of HSV-1 infected mice. Absolute numbers of TNF-α^+ **(a)**, IL-6$^+$ **(b)** and IL-1β^+ **(c)** immune cells in the skin of control DTA (blue) and Nav$_{1.8}$-DTA (red) mice 6 days pi (n=7-12 mice per group). The gating strategy for the identification of the cell subsets is shown in Figure 3b. Each dot represents data obtained for one mouse. The data are shown as mean ± SEM with p-values indicated in figure obtained by Mann-Whitney test (two-tailed).

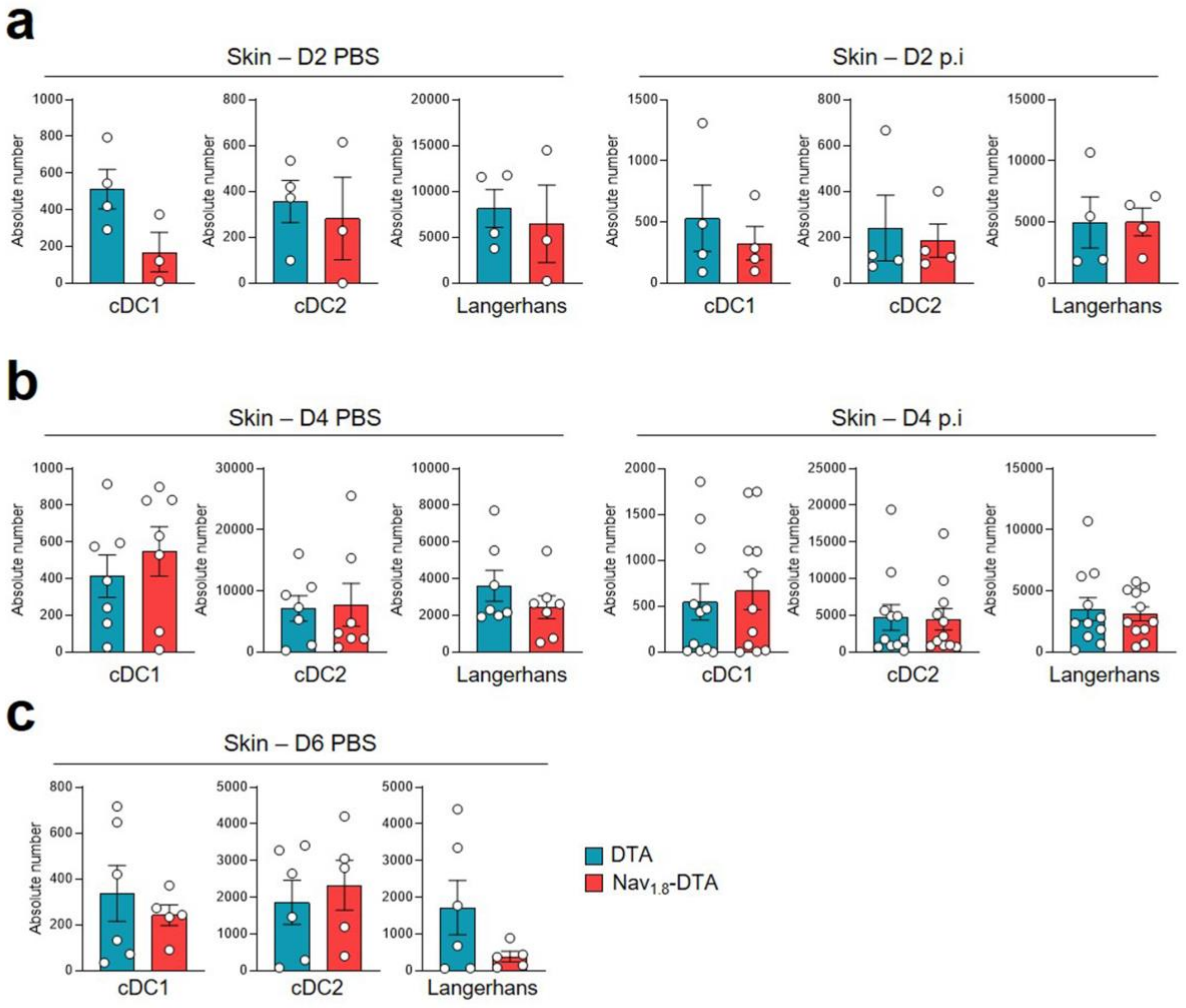

Supplementary figure 3. Analysis of DC subsets in the skin upon infection. (a, b, c) Absolute numbers of cDC1, cDC2 and LC, in the skin of mock-infected mice (scarification + PBS) or infected mice. The gating strategies used to identify the DC subsets are shown in Figure 3b. **(a)** data obtained on day 2 pi, n=4 for DTA (blue) and n=3 for $Nav_{1.8}$-DTA (red). **(b)** data obtained on day 4 pi**,** n=7 for DTA (green) and n=7 for $Nav_{1.8}$-DTA (red). **(c)** data obtained on day 6 post-scarification, n=6 for DTA (blue) and n=5 for $Nav_{1.8}$-DTA (red) for control DTA and $Nav_{1.8}$-DTA mice. The data are presented as mean ± SEM. Each dot represents data obtained for one mouse.

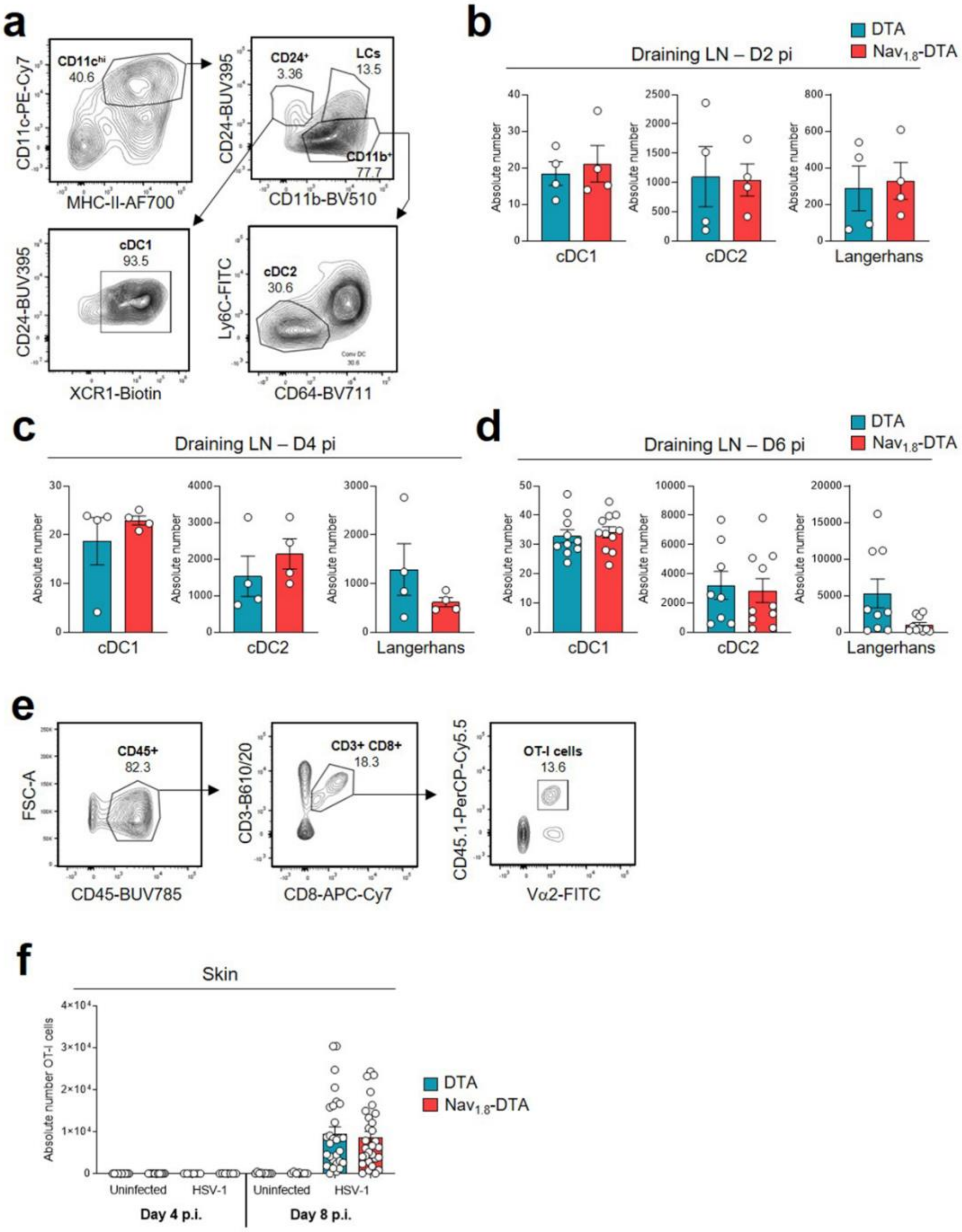

Supplementary figure 4. Analysis of DC subsets and T cell responses upon infection. (a) Gating strategy to identify DC subsets in skin draining LN. LC were defined as CD24$^+$ CD11b$^+$ DC, cDC1 as CD11b$^-$ CD24$^+$ XCR1$^+$ DC and cDC2 as CD24$^-$ CD11b$^+$ Ly6C$^-$ CD64$^-$ DC. **(b, c, d**) Absolute numbers of DC subsets, including cDC1, cDC2 and LC, in brachial draining LN of infected control DTA and $Nav_{1.8}$-DTA mice mice on day 2 (n=4 per group) **(b)**, day 4 (n=4 per group) **(c)** or day 6 pi (n=8-10 per group) **(d)**. **(e)** Gating strategy to identify OT-I T cells by flow cytometry analysis. OT-I T cells were defined as CD45.1+ CD3+ CD8+ Vα2+ cells. **(f)** Absolute numbers of OT-I T cells in the skin at the indicated time points (n=7-29 per group). The data were obtained in least three independent experiments. Each dot represents the data obtained for one mouse. The mean ± SEM are shown.

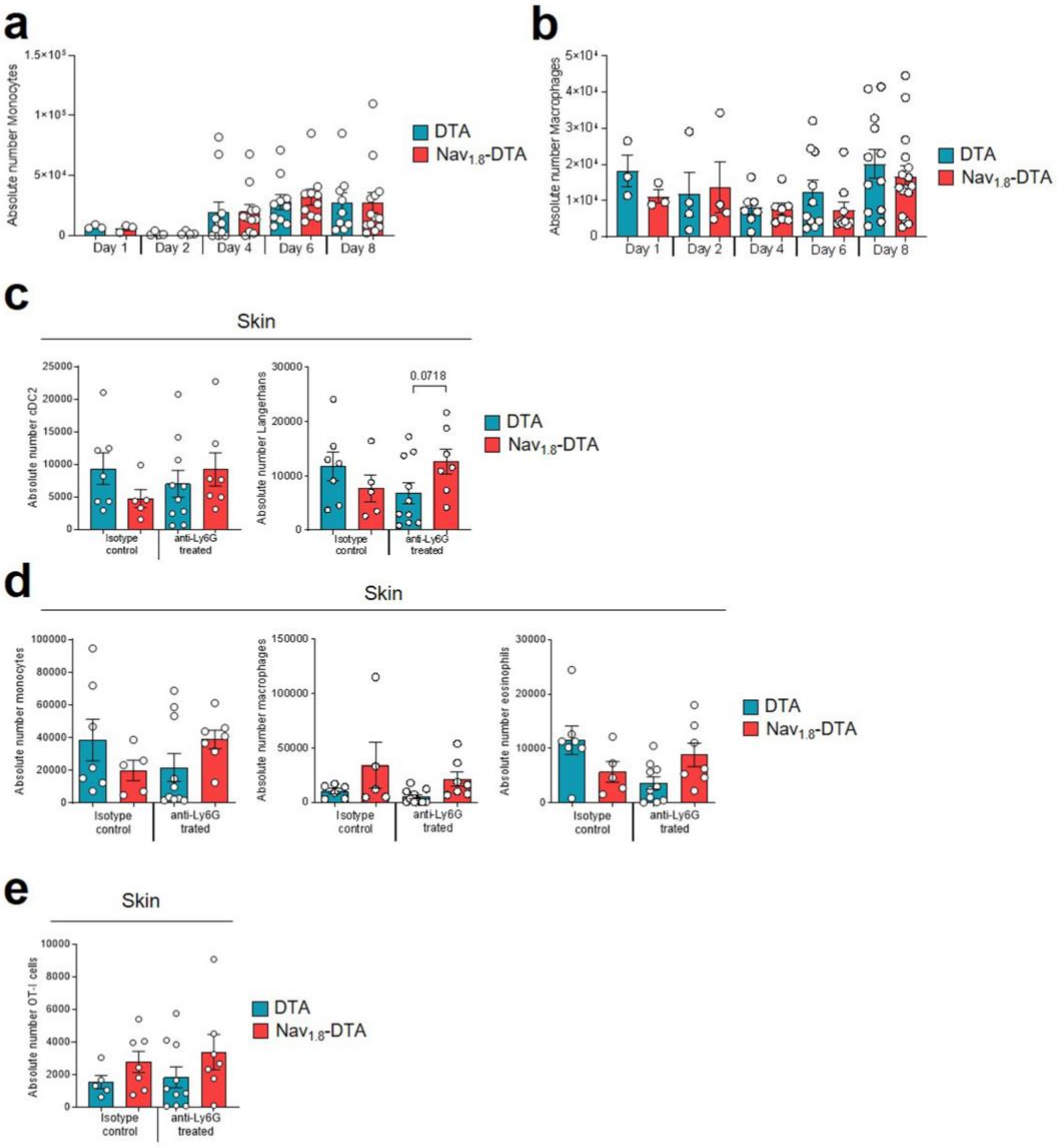

Supplementary figure 5. Analysis of myeloid and lymphoid cell responses to infection. (a, b) Longitudinal follow-up of the absolute numbers of monocytes **(a)** and macrophages (n=3-16 mice per group) **(b)** in the skin of HSV-1-infected control DTA (blue) and $Nav_{1.8}$-DTA (red) mice. The gating strategy used to identify these cell subsets is shown in Figure 3b. **(c)** Skin DC were analyzed by flow cytometry. $CD11c^{hi}$ $MHC\text{-}II^{+}$ DCs were classified into subsets on the basis of their expression of the CD24, CD11b, XCR1, Ly6C and CD64 markers as shown in Supplementary Figure 4a. The absolute numbers of cDC2 and LC in the skin are shown for 8 days pi (n=5-10 mice per group). Data are presented as mean ± SEM. p-value were obtained by one-way ANOVA followed by Sidak's multiple comparison test. **(d)** Absolute number of monocytes, macrophages and eosinophils in the skin of HSV-1-infected control DTA (blue) and $Nav_{1.8}$-DTA (red) mice treated with anti-Ly6G antibodies or isotype control (n=5-10 mice per group). The gating strategy used to identify these cell subsets is shown in Figure 3b. **(e)** Absolute numbers of OT-I T cells in the skin of control DTA and $Nav_{1.8}$-DTA mice treated with the isotype control or anti-Ly6G antibody on day 8 pi (n=5-10 mice per group). The gating strategy to identify OT-I T cells is shown in the Supplementary figure 4e. The results from three independent experiments are shown and presented as mean ± SEM.

Investigator 1

	DTA mice - HSV-1														
	Experiment 1				Experiment 2			Experiment 3			Experiment 4			Experiment 5	
D2	0.5	1.1	3.9	1.4	0	0	0	0.6	3.3	0.8	1.1	0.6	1.5	0.7	4.7
D3	1.2	1.6	3.4	2.1	2.1	1.2	8.4	3.3	3.3	11.2	3.6	4.7	7.1	1.1	5.9
D4	5.1	2	2.5	1.9	2.9	1.1	6.8	48.2	48.2	45.2	2.4	5.2	6.4	25.1	8.1
D5	1.3	12.8	6.4	13.1	3.2	1.6	4.6	17.4	17.4	41.2	2.8	3.9	7	24.4	34.4
D6	6.6	6.1	5.2	4.3	3.1	1.4	6	9.3	9.3	25	1.8	4.9	2.6	19.6	29.1
D7	6.3	1.9	3.5	5.2	2.5	0.8	3.6	4.3	4.3	10.3	0.7	1.2	1	2.7	14.1
D8								0.3	0.3	10.4	0.2	1	0.4	0.5	5

	$Nav_{1.8}$-DTA mice - HSV-1													
	Experiment 1				Experiment 2			Experiment 3			Experiment 4			Experiment 5
D2	1.6	1.1	4.3	2.1	0	0	4	0.9	0.4	1.1	0.7	1	1.3	1.2
D3	11.5	21.3	9.2	2.1	6.6	11.2	137.9	14	12.6	3.2	10.2	15.3	5.5	38.2
D4	19.45	208.6	11.65	91.9	89.5	59.5	145.8	86.3	22.8	110.5	31.5	63.1	4	66.7
D5	27.4	123.3	14.1	104.3	71	87.7	113.4	82.4	23.3	78.7	60.2	56	5.8	85
D6	30.2	81	9.4	74.9	48.6	49.8	117.5	75.6	20.8	47.2	37	44.7	11.2	78
D7	21	56.8	7.8	47	29.1	33.8	74.3	12.3	15.7	39.2	12.5	33.7	6	91
D8								6.7	15	12.9	5.1	15.6	1.2	69

	DTA mice - PBS				
	Experiment 1	Experiment 2	Experiment 3	Experiment 4	Experiment 5
D2	1.6	0.6	0.9	0.7	0.5
D3	1.2	0.6	1.7	1.1	0.7
D4	1.8	5.2	38.3	0.7	1.2
D5	100	4.1	34.4	0	1.3
D6	29.1	0	35.5	0	0.7
D7	0.4	0	0	0	1.7
D8			0	0	0

	$Nav_{1.8}$-DTA mice - PBS				
	Experiment 1	Experiment 2	Experiment 3	Experiment 4	Experiment 5
D2	1.5	0.8	2.8	0.6	0.6
D3	2.1	0.8	4	4.1	0.6
D4	3.3	7.1	47.8	4.1	1.4
D5	6.7	8.6	40.7	3	40.2
D6	2	5.6	19.8	4	24.1
D7	1.8	1.2	16.6	9.1	13.7
D8			8.4	3.7	3.1

Investigator 2

	DTA mice - HSV-1				
	Experiment 6		Experiment 7		
D2	0	0	0	0	0
D3	1.6	1.4	1.2	3	2.8
D4	1.1	2	2.1	3.7	3.6
D5	2	6.3	4.1	4	2.4
D6	1.3	5.1	3	2.3	2.4

	$Nav_{1.8}$-DTA mice - HSV-1				
	Experiment 6		Experiment 7		
D2	1	0	1.2	0	0
D3	1.4	3.5	1.5	3.3	2.6
D4	6.2	6.6	17.6	5.8	4.5
D5	30.3	6.4	23.1	12.6	13.7
D6	27.1	12.6	9.2	10.5	2.2

	DTA mice - PBS			
	Experiment 6		Experiment 7	
D2	0	0	0	0
D3	0.8	0.3	2.1	0.8
D4	1.2	0.8	2.6	2
D5	1.4	1.1	1.8	0.6
D6	0.8	0	0.9	0.4

	$Nav_{1.8}$-DTA mice - PBS		
	Experiment 6		Experiment 7
D2	0	0	0
D3	1.7	0.7	1.5
D4	2.3	1.1	0.4
D5	1.1	1.3	0.4
D6	0	0.7	0

Supplementary Table 1: Evolution of lesion size. The size of the lesion was monitored from D2 to D8 after scarification (PBS condition) or HSV-1 infection. The raw data obtained in 7 independent experiments performed by two different experimenters are shown. These data correspond to the data included in the Figures 1d and 1e. All the analysis of lesion size were done by the same investigator using photographs. The investigator was blinded to the genotype of each mouse during the analysis.
