# Supplementary material for: Nociceptive sensory neurons promote CD8 T cell responses to HSV-1 infection
Source: Nat Commun. 2021 May 18;12:2936. doi: 10.1038/s41467-021-22841-6 (PMC8131384; doi:10.1038/s41467-021-22841-6)
Supplement: Supplementary file 3 — Reporting Summary [file 41467_2021_22841_MOESM3_ESM.pdf]

nature research

Corresponding author(s): Sophie Ugolini

Last updated by author(s): Mar 26, 2021

Reporting Summary

Nature Research wishes to improve the reproducibility of the work that we publish. This form provides structure for consistency and transparency in reporting. For further information on Nature Research policies, see Authors & Referees and the Editorial Policy Checklist .

Statistics

For all statistical analyses, confirm that the following items are present in the figure legend, table legend, main text, or Methods section.

n/a	Confirmed	
☐	☒	The exact sample size (*n*) for each experimental group/condition, given as a discrete number and unit of measurement
☐	☒	A statement on whether measurements were taken from distinct samples or whether the same sample was measured repeatedly
☐	☒	The statistical test(s) used AND whether they are one- or two-sided<br>*Only common tests should be described solely by name; describe more complex techniques in the Methods section.*
☒	☐	A description of all covariates tested
☐	☒	A description of any assumptions or corrections, such as tests of normality and adjustment for multiple comparisons
☐	☒	A full description of the statistical parameters including central tendency (e.g. means) or other basic estimates (e.g. regression coefficient) AND variation (e.g. standard deviation) or associated estimates of uncertainty (e.g. confidence intervals)
☐	☒	For null hypothesis testing, the test statistic (e.g. *F*, *t*, *r*) with confidence intervals, effect sizes, degrees of freedom and *P* value noted<br>*Give P values as exact values whenever suitable.*
☒	☐	For Bayesian analysis, information on the choice of priors and Markov chain Monte Carlo settings
☒	☐	For hierarchical and complex designs, identification of the appropriate level for tests and full reporting of outcomes
☒	☐	Estimates of effect sizes (e.g. Cohen's *d*, Pearson's *r*), indicating how they were calculated

Our web collection on statistics for biologists contains articles on many of the points above.

Software and code

Policy information about availability of computer code

Data collection	BD FACS Diva 8.0.1 software was used for cytofluorimetric data collection.
Data analysis	GraphPad Prism8 was used for the statistical analyses. FlowJo10.7 was used for the analyses of the FACS data. ZEN 2.3 software and ImageJ 1.53c was used for pictures analyses. FCAP software TM V3 was used for cytometric bead array analysis.

For manuscripts utilizing custom algorithms or software that are central to the research but not yet described in published literature, software must be made available to editors/reviewers. We strongly encourage code deposition in a community repository (e.g. GitHub). See the Nature Research guidelines for submitting code & software for further information.

Data

Policy information about availability of data

All manuscripts must include a data availability statement. This statement should provide the following information, where applicable:

- Accession codes, unique identifiers, or web links for publicly available datasets
- A list of figures that have associated raw data
- A description of any restrictions on data availability

The data that support the findings of this study are available from the corresponding author upon request.

Field-specific reporting

Please select the one below that is the best fit for your research. If you are not sure, read the appropriate sections before making your selection.

☒ Life sciences ☐ Behavioural & social sciences ☐ Ecological, evolutionary & environmental sciences

Life sciences study design

All studies must disclose on these points even when the disclosure is negative.

Sample size	No sample size calculation was performed, but a reasonable sample size was chosen to ensure adequate reproducibility of results and was based on our previous studies.
Data exclusions	No data was excluded.
Replication	Experiments were replicated several times, as indicated in each figure legends, with reproducible results.
Randomization	Mice were assigned to experimental groups according to sex and age. Only female mice were used in this study.
Blinding	In the in vivo experiments the investigators were not always blinded to the genotype of the experimental groups.

Reporting for specific materials, systems and methods

We require information from authors about some types of materials, experimental systems and methods used in many studies. Here, indicate whether each material, system or method listed is relevant to your study. If you are not sure if a list item applies to your research, read the appropriate section before selecting a response.

Materials & experimental systems

n/a	Involved in the study	
☐	☒	Antibodies
☐	☒	Eukaryotic cell lines
☒	☐	Palaeontology
☐	☒	Animals and other organisms
☒	☐	Human research participants
☒	☐	Clinical data

Methods

n/a	Involved in the study	
☒	☐	ChIP-seq
☐	☒	Flow cytometry
☒	☐	MRI-based neuroimaging

Antibodies

Antibodies used	The following antibodies were used for flow cytometry: CD11b (BV510, M1/70, 562950,1/800), , Ly6C (BV421, AL-21, 562727, 1/300), CD4 (FITC, H129.19, 553651, 1/100), CD11c (BUV395, N418, 744180, 1/200), CD24 (BUV737, M1/69, 612832, 1/1000), betaTCR (BV711, H57-597, 563135, 1/100), CD8 (APC, 53-6.7, 561093, 1/300 or APC-Cy7, 53-6.7, 560182, 1/100), CD19 (PE-CF594, 1D3, 562291, 1/200), NK1.1 (PE-CF594, PK136, 562864, 1/100), Ly6G (PE-CF594, 1A8, 562700, 1/300), Siglec F (PE, E50-2440, 562068, 1/500), Va2 (FITC, B20.1, 553288, 1/700), gamma/deltaTCR (PE-Cy5, GL3, 15-5711-82, 1/500) all from BD. CD45 (BV785, 30-F11, 103149, 1/600), CD64 (BV711, X54-5/7.1, 139311, 1/300), c-kit (BV605, ACK2, 135122, 1/300), XCR1 (biotin, ZET, 148212, 1/200), GR-1 (Biotin, RB6-8C5, 108404, 1/500), Ly6G (APC-Cy7, 1A8, 127624, 1/500), MHCII (AF700, M5/114.15.2, 107622, 1/600), CD206 (APC, CO68C2, 141708, 1/400) all from biolegend. CCR2 (AF647, 475301, FAB5538R, 1/25) from R&D and CD3 (B610/20145-2C11, 1/300) from eBiosciences. TNFalpha (PE, MP6-XT22, BD, 561063, 1/300), IL-6 (PE, MP5-20F3, BD, 554401, 1/100) and IL-1beta (PE, NJTEN3, 4330860, BD, 1/300).The following antibodies were used for immunofluorescence : goat anti-mouse CD45 (purified, AF114-SP, R&D systems, 1/300), rat anti-mouse Langerin (purified, eBioRMUL.2, 15237307, Fisher Scientific, 1/300), rabbit anti-mouse PGP9.5 (purified, RB-9202-P1, Fisher Scientific, 1/300), CD3 (APC, 145-2C11, 1000322, Biolegend, 1/300), goat anti-rat AF488 (Jackson immunoresearch, 112-545-003, 1/500), donkey anti-rabbit AF555 (Invitrogen, A31572) and donkey anti-goat AF647 (Jackson immunoresearch, 705-605-147, 1/500). The following antibodies were used for neutrophils depletion : rat anti-mouse Ly6G (1A8, BP0075, BioXCell, 200 mg) and rat IgG2a isotype control (2A3, BE0089, BioXCell, 200 mg).
Validation	All the antibodies used are from commercial sources and have been validated by the vendors. Validation data for species (mouse) and application (flow cytometry and in vivo injection) are available on the manufacturer's website.

Eukaryotic cell lines

Policy information about cell lines

Cell line source(s)	Vero cells (CSL) were from ATCC® (ATCC CCL-81)
Authentication	Cell lines were used after recommendation by Francis Carbone
Mycoplasma contamination	Mycoplasma test were done regularly and all test were negative for mycoplasma
Commonly misidentified lines (See ICLAC register)	No misidentified lines are used in this study.

Animals and other organisms

Policy information about studies involving animals; ARRIVE guidelines recommended for reporting animal research

Laboratory animals	Nav1.8-Cre mice were provided by Dr. Aziz Moqrich,. Heterozygous Nav1.8-Cre mice were crossed with homozygous DTA mice (B6.129P2-t(ROSA)26Sortm1(DTA)Lky/J The Jackson Laboratory JAX: 009669) to generate a 1:1 ratio of Nav1.8-Cre -DTA and DTA littermates. Nav1.8-CrexTdTomato(Nav1.8-Cre-TdTomato) mice were obtained by crossing Nav1.8-Cre mice with TdTomato mice (B6.Cg-Gt(ROSA)26Sortm14(CAG-tdTomato)Hze The Jackson Laboratory JAX: 007914). OT-I mice (ovalbumin-specific TCR-transgenic mice) (Hogquist et al., 1994) were provided by Dr. Bernard Malissen. Mice were housed under a standard 12h:12 h light-dark cycle with ad libitum access to food and water, T° 22°C +/- 1°C, 45-60% humidity. Age-matched (6-12 weeks old) and sex-matched (all the mice used were female) littermate mice were used as controls.
Wild animals	No wild animals were used.
Field-collected samples	The study did not used collected samples from the field.
Ethics oversight	Permission was granted to perform animal experiments by the institutional committee (Comité d'éthique en experimentation animale n°014) (project number: E13-055-10).

Note that full information on the approval of the study protocol must also be provided in the manuscript.

Flow Cytometry

Plots

Confirm that:

- [x] The axis labels state the marker and fluorochrome used (e.g. CD4-FITC).
- [x] The axis scales are clearly visible. Include numbers along axes only for bottom left plot of group (a 'group' is an analysis of identical markers).
- [x] All plots are contour plots with outliers or pseudocolor plots.
- [x] A numerical value for number of cells or percentage (with statistics) is provided.

Methodology

Sample preparation	Spleen, LN (axillary and brachial) and skin samples (12x12 mm punch biopsy fullthickness pieces) were cut into small fragments and placed in a collagenase/dispase/DNase digestion solution (0.2 mg/mL collagenase type IV [Sigma] 0.2 mg/ml dispase (Gibco) and 1 mg/ml DNase (Roche) in RPMI 1640 complete medium and incubated at 37°C for 1h. Spleen and lymph nodes were crushed in FACS buffer on a cell strainer with 70 microm pores (BD Biosciences) and red blood cells from the spleen were lysed in RBC lysis buffer (Invitrogen). Digestion was stopped by adding 10% RPMI 1640. The suspensions were filtered, washed and stained for FACS analysis.
Instrument	Samples were analysed in a BD LSR Fortessa X20 flow cytometer (BD Biosciences).
Software	BD FACS Diva 8.0.1 software was used for data collection and FlowJo v.10 was used for data analysis.
Cell population abundance	No sorting was used in this study. T cells were isolated and purified from LN and spleens of transgenic OT-I mice by negative selection using the Pan T Cell Isolation Kit II from Miltenyi Biotec. Cell preparations were routinely 85–95% pure, as tested by flow cytometry.
Gating strategy	Among live, single, CD45+ cells :<br>Neutrophils were gated as: Ly6G+CD11b+ cells<br>Skin monocytes were gates as: Ly6G-CD45+CD11b+Ly6C+CD206-<br>Skin macrophages were gates as: Ly6G-CD45+CD11b+Ly6C-CD64+CD206+<br>Skin eosinophils were gated as: Ly6G-CD11b+CD24+<br>Skin dendritic cells were gates as: CD11chiMHC-II+ and subdivided into subsets: Langerhans cells were gated as CD24+CD11b+ DCs, cDC1 were gated as CD11b-CD24+XCR1+ DCs and cDC2 were gated as CD11b+CD24-DCs.<br>Skin mast cells were gated as : CD11b+ CD45+ c-kit+<br>Skin CD4+ T cells were gated as : CD11b- CD45+ TCRgamma/delta- CD4+<br>Skin gamma/delta T cells were gated as : CD11b- CD45+ TCR gamma/delta+ CD4-<br>OT-I T cells were gated as : CD3+ CD8+ CD45.1+ Va2+

- [x] Tick this box to confirm that a figure exemplifying the gating strategy is provided in the Supplementary Information.
